# Anti‐Siglec‐15 Antibody Prevents Marked Bone Loss after Acute Spinal Cord Injury‐Induced Immobilization in Rats

**DOI:** 10.1002/jbm4.10825

**Published:** 2023-09-27

**Authors:** Yuanzhen Peng, Solomon Langermann, Priyanka Kothari, Linda Liu, Wei Zhao, Yizhong Hu, Zihao Chen, Mariana Moraes de Lima Perini, Jiliang Li, Jay Cao, X. Edward Guo, Lieping Chen, William A. Bauman, Weiping Qin

**Affiliations:** ^1^ Spinal Cord Damage Research Center, James J. Peters Veteran Affairs Medical Center Bronx New York USA; ^2^ NextCure, Inc Beltsville Maryland USA; ^3^ Department of Biomedical Engineering Columbia University New York New York USA; ^4^ Department of Biotechnology Brown University Providence Rhode Island USA; ^5^ School of Science, Indiana University Purdue University Indianapolis Indiana USA; ^6^ USDA‐ARS Grand Forks Human Nutrition Research Center Grand Forks North Dakota USA; ^7^ Cancer Research, Immunobiology and Medicine, The Yale University School of Medicine New Haven Connecticut USA; ^8^ Departments of Medicine Rehabilitation and Human Performance, Icahn School of Medicine at Mount Sinai New York New York USA; ^9^ Rehabilitation and Human Performance, Icahn School of Medicine at Mount Sinai New York New York USA

**Keywords:** BONE FORMATION, BONE RESORPTION, IMMOBILIZATION, SIGLEC‐15, SPINAL CORD INJURY

## Abstract

Rapid and extensive sublesional bone loss after spinal cord injury (SCI) is a difficult medical problem that has been refractory to available interventions except the antiresorptive agent denosumab (DMAB). While DMAB has shown some efficacy in inhibiting bone loss, its concurrent inhibition of bone formation limits its use. Sialic acid‐binding immunoglobulin‐like lectin (Siglec)‐15 is expressed on the cell surface of mature osteoclasts. Anti‐Siglec‐15 antibody (Ab) has been shown to inhibit osteoclast maturation and bone resorption while maintaining osteoblast activity, which is distinct from current antiresorptive agents that inhibit the activity of both osteoclasts and osteoblasts. The goal of the present study is to test a Siglec‐15 Ab (NP159) as a new treatment option to prevent bone loss in an acute SCI model. To this end, 4‐month‐old male Wistar rats underwent complete spinal cord transection and were treated with either vehicle or NP159 at 20 mg/kg once every 2 weeks for 8 weeks. SCI results in significant decreases in bone mineral density (BMD, −18.7%), trabecular bone volume (−43.1%), trabecular connectivity (−59.7%), and bone stiffness (−76.3%) at the distal femur. Treatment with NP159 almost completely prevents the aforementioned deterioration of bone after SCI. Blood and histomorphometric analyses revealed that NP159 is able to greatly inhibit bone resorption while maintaining bone formation after acute SCI. In ex vivo cultures of bone marrow cells, NP159 reduces osteoclastogenesis while increasing osteoblastogenesis. In summary, treatment with NP159 almost fully prevents sublesional loss of BMD and metaphysis trabecular bone volume and preserves bone strength in a rat model of acute SCI. Because of its unique ability to reduce osteoclastogenesis and bone resorption while promoting osteoblastogenesis to maintain bone formation, Siglec‐15 Ab may hold greater promise as a therapeutic agent, compared with the exclusively antiresorptive or anabolic agents that are currently used, in mitigating the striking bone loss that occurs after SCI or other conditions associated with severe immobilization. © 2023 The Authors. *JBMR Plus* published by Wiley Periodicals LLC on behalf of American Society for Bone and Mineral Research. This article has been contributed to by U.S. Government employees and their work is in the public domain in the USA.

## Graphical abstract

Rapid and extensive sublesional bone loss after spinal cord injury (SCI) is a difficult medical problem that has been refractory to available interventions except the antiresorptive agent denosumab (DMAB). While DMAB has shown some efficacy in inhibiting bone loss, its concurrent inhibition of bone formation limits its use. In this study, we demonstrated that treatment with anti‐Siglec‐15 antibody (NP159) almost fully prevents sublesional loss of bone mineral density and metaphysis trabecular bone volume and preserves bone strength in a rat model of acute SCI. Because of its unique ability to reduce osteoclastogenesis and bone resorption while promoting osteoblastogenesis to maintain bone formation, Siglec‐15 Ab may hold greater promise as a therapeutic agent, compared with the exclusively antiresorptive or anabolic agents that are currently used, for mitigating the striking bone loss that occurs after SCI or other conditions associated with severe immobilization.

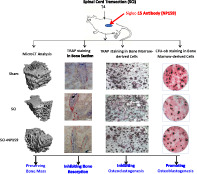

## Introduction

Of the total noninstitutionalized population in the United States in 1988, 3.8% (8.8 million people) were estimated not to be able to perform any major activity.^[^ ^1^ ^]^ Immobilization osteoporosis represents a wide spectrum of conditions and disorders. A few representative examples encompass a range of rates of bone loss including individuals confined to bed rest (e.g., stroke, poliomyelitis) at 0.1% per week, microgravity at 0.25% per week, and motor‐complete spinal cord injury (SCI) at 1% per week at select skeletal regions during the initial months.^[^ ^2^, ^3^, ^4^, ^5^, ^6^ ^]^ In contrast, postmenopausal osteoporosis, a nonimmobilizing condition associated with bone loss with heightened awareness in the medical community, has a bone loss of 3% to 5% per year when not prescribed antiresorptive medication.^[^ ^7^ ^]^ Although efficacious strategies have been developed for other forms of osteoporosis (e.g., sex‐hormone‐deficient, glucocorticoid‐induced, nutritional deficiency osteoporosis), the ability to maintain bone when load is acutely reduced is, at present, far from clinically satisfactory.^[^ ^8^ ^]^

In the United States, there are ~288,000 people with SCI, with ~17,700 new cases of SCI occurring annually.^[^ ^9^, ^10^ ^]^ The impetus for the present study is to develop novel therapeutics to treat the severe loss of bone that occurs in regions of the unloaded skeleton affected by paralysis after SCI. Over the first couple of years after SCI, 50% to 60% of bone mineral density (BMD) may be lost at the epiphyseal and metaphyseal regions of the long bones of the lower extremities.^[^ ^11^ ^]^ Almost immediately after SCI, bone resorption and the associated calcium excretion are greatly increased. Histologic measures of bone biopsy samples revealed that bone formation was reduced in individuals with SCI.^[^ ^12^ ^]^ Because SCI causes rapid and extensive loss of sublesional bone, those with SCI have an increased risk of fracture.^[^ ^8^, ^13^, ^14^ ^]^ Bone may be lost in trabecular regions of the sublesional skeleton at a rate as great as ~1% per week for the first year after SCI and continue to be lost at a rapid rate over the next year or so, while cortical bone is lost by endocortical resorption at an increased rate for at least the initial 7 years or longer after paralysis, depending on the region.^[^ ^15^, ^16^, ^17^ ^]^ The rate at which bone is lost after SCI is greater than 10 times that of postmenopausal osteoporosis^[^ ^18^ ^]^ and is more severe than other types of disuse osteoporosis, such as microgravity^[^ ^2^ ^]^ and prolonged bed rest.^[^ ^6^ ^]^ It should be noted that central nervous system control of skeletal muscle is maintained in persons under conditions of microgravity and bed rest, as well as in animals with tail suspension, but is disrupted to varying degrees after SCI depending on the degree of neurological injury. In contrast to postmenopausal osteoporosis, a condition that is largely due to estrogen deficiency, rapid and extensive bone loss after SCI is mainly attributed to immediate, severe, and irreversible unloading of bone, which is complicated further by other associated pathological changes (e.g., neurological, hormonal, metabolic, and inflammatory disorders).^[^ ^10^, ^14^, ^18^ ^]^

The prevalence of fractures in persons with SCI is reported to be as high as 25% to 46%,^[^ ^19^ ^]^ and fractures occur with relatively minor stress or trauma.^[^ ^20^ ^]^ The average hospital stay following fracture in a patient with SCI is 35 days, seven times longer than those for admissions without fractures.^[^ ^21^, ^22^ ^]^ The prolonged hospital stay is, in part, due to the fact that the majority of these patients experience local, general, or orthopedic complications.^[^ ^23^ ^]^ Furthermore, nearly four out of five patients are not allowed to perform unassisted transfers for an average of 65 days, adding substantially to caregiver time and cost.^[^ ^23^ ^]^ Importantly, a heightened risk of fracture due to bone loss may preclude participation in activity‐based rehabilitation or the use of promising exoskeletal devices or modalities of spinal cord stimulation for ambulation and may prevent the ability to participate in future advances to be made in neurorepair. Given the lack of any practical intervention available to safely inhibit bone loss or restore a sufficient fraction of the bone loss in people with SCI, there is a clear unmet need to develop an efficacious treatment to improve sublesional bone integrity, more specifically a therapeutic that improves bone integrity and function at the distal femur and proximal tibia, which are the skeletal sites at highest risk of fracture.

Sialic acid‐binding immunoglobulin‐like lectin (Siglec)‐15, a member of cell‐surface receptors, regulates the functions of cells in the innate and adaptive immune system through glycan recognition.^[^ ^24^, ^25^ ^]^ Recent in vitro studies revealed upregulated Siglec‐15 expression in differentiated osteoclasts.^[^ ^25^, ^26^ ^]^ Among the known Siglecs expressed in myeloid lineage cells, only Siglec‐15 was upregulated by receptor activator of nuclear factor kB ligand (RANKL) in mouse bone marrow macrophages.^[^ ^25^ ^]^ In response to RANKL, Siglec‐15 is shown to interact with the adapter protein DAP12 and induce Akt activation when clustered on the osteoclast cell surface for osteoclastogenesis.^[^ ^27^ ^]^ An antibody has been generated against Siglec‐15 that inhibits osteoclast fusion and maturation but does not inhibit formation of mononuclear preosteoclasts. The resultant preosteoclasts are able to support osteoblast activity and bone formation and, thus, play an important role in decoupling bone resorption and formation.^[^ ^27^, ^28^ ^]^ Recent work demonstrated that the Siglec‐15 Ab was able to reduce bone resorption while maintaining bone formation in ovariectomized (OVX) rats and monkeys^[^ ^29^ ^]^ and increase BMD and bone strength of cortical bone in OVX cynomolgus monkeys.^[^ ^30^ ^]^ A phase I clinical trial in postmenopausal women indicated that a single dose of the Siglec‐15 Ab resulted in sustained decreases in bone biomarkers of resorption while having a modest effect on bone biomarkers of formation. The observed efficacy seen with a single dose is associated with a good safety profile: there were no major toxicities or drug‐related serious adverse events, symptomatic hypocalcemia, or clinically meaningful changes in numbers of circulating leukocyte subtypes or antibody‐mediated hyperstimulation of cytokines.^[^ ^31^ ^]^ More recently, Cao and colleagues demonstrated that a novel Siglec‐15 Ab (NP159) could block osteoclast maturation and osteoclastogenesis and increase bone volume in OVX mice.^[^ ^32^ ^]^ Of note, Siglec‐15 Ab‐mediated inhibition of osteoclastogenesis by NP159 is associated with significant increases in tartrate‐resistant acid phosphatase (TRACP)‐positive preosteoclasts (mononuclear cells), resulting in an increased production of platelet‐derived growth factor‐BB (PDGF‐BB) from preosteoclasts that can promote the recruitment of osteoblasts, thereby leading to anabolic activity to increase bone formation and promote facture healing.^[^ ^32^ ^]^

These unique and favorable features differentiate Siglec‐15 Abs from current antiresorptive agents (e.g., bisphosphonate [BP] and anti‐RANKL Ab [denosumab, DMAB]) that inhibit the activity of both osteoclasts and osteoblasts, as well as from other bone anabolic agents (e.g., teriparatide, abaloparatide, and romosozumab [ROMO]) that have major safety concerns with long‐term use. For these reasons, Siglec‐15 Abs hold greater promise to improve skeletal health in individuals with SCI than that of currently available antiresorptive or anabolic bone agents. At present, the efficacy of Siglec‐15 Ab is not known in immobilization‐induced bone loss of any etiology, including SCI. As is currently understood, an agent that is effective and safe in other conditions of bone loss may not offer the same degree of efficacy and safety profile to persons with SCI. Here we assess whether the anti‐Siglec‐15 mAb NP159 has therapeutic potential in SCI.

The present study in rodent models that is described herein represents the first effort to evaluate the ability of a novel strategy to markedly reduce bone loss after acute SCI by inhibiting the biological activity of Siglec‐15. We hypothesize that Siglec‐15 Ab can serve not only as a novel antiresorptive but also as a unique bone formation agent to improve bone integrity after SCI. In this study, an established rat model of bone loss following complete spinal cord transection^[^ ^10^, ^33^, ^34^, ^35^, ^36^, ^37^, ^38^, ^39^, ^40^, ^41^, ^42^ ^]^ was used to investigate the skeletal effects of NP159 treatment for 8 weeks when initiated immediately after injury. The effects of NP159 were examined on bone mass and architecture, blood and histomorphometric indices of bone formation and resorption, and osteoclastogenic and osteoblastogenic potential of bone marrow progenitor cells, as well as on gene expression profiling following treatment.

## Materials and Methods

### Animals, surgery, drug administration, and tissue collection

All animals were housed in a temperature‐ and humidity‐controlled room providing a 12:12‐hour day:night cycle and fed food and water ad libitum. All animal studies were approved by the Institutional Animal Care and Use Committee at the James J. Peters Veterans Affairs Medical Center and conformed to all guidelines and regulations for the protection of the welfare of animal subjects. In this study, three groups of animals were studied: spinal cord‐transected treated with vehicle (normal saline) (SCI animals, *N* = 15), spinal cord‐transected treated with human Siglec‐15 Ab monoclonal antibody (NP159) (SCI + NP159 animals, *N* = 13), and a sham‐SCI group (sham animals, *N* = 14). Spinal cord transection surgery was performed as previously described.^[^ ^10^, ^33^, ^34^, ^35^, ^36^, ^40^, ^42^, ^43^ ^]^ Briefly, 4‐month‐old male Wistar rats (~350 g) were purchased from Charles River (Wilmington, Massachusetts) were anesthetized by inhalation of isofluorane (3% to 5%), and hair was removed with a clipper. Skin over the back was cleaned with betadine and isopropyl alcohol. After making a midline incision, the spinal cord at the interspace between the third and fourth vertebral bodies was visualized by laminectomy, and the spinal cord was transected with microscissors. The space between transected ends of the spinal cord was filled with surgical sponge, and the wound was closed in two layers with suture.

Urine was voided at least three times daily until automaticity of the bladder developed, then at least once a day as needed. Baytril was administered for the first 3 to 5 days postoperatively, and then as indicated for cloudy or bloody urine or for overt wound infection. Sham animals received an identical surgery, including a laminectomy, except that the spinal cord was not cut. Immediately after spinal cord transection, SCI rats were injected peritoneally with saline or NP159 at 20 mg/kg^[^ ^44^, ^45^, ^46^ ^]^ once every 2 weeks for 8 weeks. We chose 8 weeks of treatment with NP159 because bone loss due to SCI is far greater than that observed in other types of osteoporosis resulting from aging and ovariectomy.^[^ ^45^, ^46^ ^]^

On day −6 and day −2 prior to euthanasia, the animals were labeled with fluorochromes by subcutaneous injection of calcein (10 mg/kg body weight) and xylenol orange (90 mg/kg body weight) for dynamic histomorphometric analysis. Eight weeks after NP159 was started, animals were anesthetized with isoflurane inhalation followed by transection of the aorta. The tibia and femur were removed with the knee joint intact after carefully separating bone from muscle and connective tissue, and the lumbar vertebrae was also collected. The left tibia and femur were stored in 4% paraformaldehyde for 48 hours and then kept at 70% ethanol at 4 C° for micro–CT (μCT) (*N* = 10 per group) and histomorphometric analysis (*N* = 6 or 7 per group). The right tibia and femur (*N* = 4 or 5 per group) were placed in ice‐cold Minimum Essential Alpha Medium (α‐MEM) and then immediately processed for bone marrow cell cultures.

### BMD assessed by dual‐energy X‐ray absorptiometry

Areal BMD measurements were performed using a small‐animal dual‐energy X‐ray absorptiometer (Lunar Piximus, WI, USA), as previously described.^[^ ^10^, ^33^, ^34^, ^35^, ^40^, ^47^, ^48^ ^]^ Hind limbs were positioned on the dual‐energy X‐ray absorptiometer platform with the knee flexed at an angle of 135°, and dual‐energy X‐ray absorptiometry images were acquired with Lunar Pixmus software. The instrument was calibrated with a phantom following the procedures recommended by the manufacturer on each day of use prior to analysis of experimental samples. The metaphysis of the distal femur and proximal tibia were selected as regions of interest (ROIs). The coefficient of variation for the repeated measurements for the ROIs was ~1.5%.

### μCT analysis of bone microarchitecture

Volumetric BMD and bone architecture of the distal femur were assessed by a Scanco μCT scanner (μCT‐40; Scanco Medical AG, Switzerland) at 16 mm isotropic voxel size, as previously described^[^ ^10^, ^34^, ^35^, ^36^, ^40^, ^42^ ^]^ and in greater detail in the Supplemental Materials. Image reconstruction and three‐dimensional (3D) quantitative analysis were performed using software provided by Scanco. Scans were initiated at the growth plate and moved proximally for a total of approximately 300 slices. A ROI consisting of 100 slices beginning 0.5 mm proximal to the growth plate and continuing in a proximal direction were included in the analysis. Mechanical properties at the distal femur trabecular bones were estimated from micro‐finite element analysis (μFEA) following the manufacturer's recommended procedures, as previously described^[^ ^49^ ^]^.^[^ ^35^, ^36^, ^50^, ^51^, ^52^ ^]^ Briefly, μFEA models were produced by converting each bone voxel to an eight‐node brick element. Bone tissue was subjected to applied uniaxial compression, with an elastic modulus of 15 GPa and Poisson's ratio of 0.3 for each element. A linear elastic analysis was used to estimate bone stiffness. Standard nomenclature and methods for bone morphometric analysis were employed.^[^ ^10^ ^]^

### Bone histomorphometric analyses

Dynamic histomorphometry was performed for fluorochrome‐based determination of rates of bone formation. For this purpose, 6‐μm sections embedded in methyl methacrylate plastic were cut using a Reichert‐Jung sledge microtome. Xylenol orange and calcein were visualized by fluorescent microscopy and the distance between labeled layers will be used as a measure of the rate of bone formation as determined by morphometry software.^[^ ^53^, ^54^ ^]^ Static histomorphometry was performed to quantify the osteoclast number and activity. In this study, TRACP stain was used to specifically label osteoclasts in deplastified distal femur sections, and slides were counterstained with hematoxylin and eosin (H&E), as described previously.^[^ ^47^, ^54^ ^]^ Osteoclasts were measured under bright field microscopy using an OLYMPUS microscope with the OsteoMeasure™ system. See Supplemental Materials for experimental procedures in greater detail.

### ELISA assays

Serum C‐terminal telopeptide of type I collagen (CTX) levels were measured using a RatLaps^TM^ enzyme‐immunoassay kit from Immunodiagnostic Systems (Fountain Hills, AZ, USA). Serum levels of P1NP were determined using a rat P1NP ELISA kit (MyBioSource). Serum concentrations of osteocalcin were measured using a rat osteocalcin immunoassay kit (Alfa Aesar). All samples were assayed in duplicate, following the manufacturer's protocols.

### Ex vivo osteoclastogenesis and osteoblastogenesis assays

Procedures for osteoblast and osteoclast formation from bone marrow stromal and hematopoietic progenitor cells were performed, as previously described,^[^ ^10^, ^33^, ^34^, ^40^, ^47^, ^48^ ^]^ and are described in greater detail in the Supplemental Materials. Briefly, to study osteoclast cells, bone marrow cells were isolated from the femora and tibiae in a‐MEM. Marrow cells were rinsed and resuspended in α‐MEM then seeded into wells using an equal number of cells in each well and cultured for 2 days in α‐MEM supplemented with human macrophage colony‐stimulating factor (M‐CSF; 5 ng/mL). The nonadherent cells were collected and purified by Ficoll‐Plus (Amersham Pharmacia Biotech Inc., Arlington Heights, IL, USA) then seeded into wells, again with an equal number of cells per plate, and incubated in α‐MEM containing M‐CSF (30 ng/mL) and RANKL (60 ng/mL) for 5 days, followed by total RNA extraction using the TRizol reagent. To study osteoblast cells, cells were flushed from the marrow cavity with α‐MEM and seeded into tissue culture wells; the harvested bone marrow cells were cultured in α‐MEM supplemented with 15% preselected FCS (Hyclone, Logan, UT, USA) and ascorbic acid‐2‐phosphate (1 mM). Recruitment of marrow stromal cells to the osteoblast lineage was assessed at 10 days of culture by extraction of total RNA with the TRizol reagent.

### RNA extraction from bone marrow cultures and quantitative PCR

Total RNAs were extracted from bone marrow cell cultures using the TRI reagent (Sigma‐Aldrich). One microgram of total RNA was used to synthesize the first strand cDNA by the High‐Capacity cDNA Reverse Transcription Kit (Applied Biosystems). Real‐time PCR determination of mRNA levels was performed in the ViiA7 system (Applied Biosystems), as described previously.^[^ ^10^, ^33^, ^34^, ^40^, ^47^, ^48^ ^]^ Relative expression levels were calculated using the 2^−ΔΔCt^ method with 18S RNA as an internal control.^[^ ^55^ ^]^ Additional details regarding procedures performed are provided in Supplemental Materials.

### Statistics

Standard power analyses were used to determine the requisite minimum number of animals to ensure sufficient statistical power, as described.^[^ ^56^ ^]^ Data are expressed as mean ± SEM; the number of independent samples (*n*) is provided in the legend of each figure. The statistical significance of differences among means was tested using one‐way ANOVA and a Newman–Keuls test post hoc to determine the significance of differences between individual pairs of means using a *p*‐value of 0.05 as the cutoff for significance. Statistical calculations were performed using Prism 4.0c (GraphPad Software, La Jolla, CA, USA).

## Results

### Siglec‐15 antibody prevented marked bone loss after acute motor‐complete SCI In rats

**Fig. 1:**
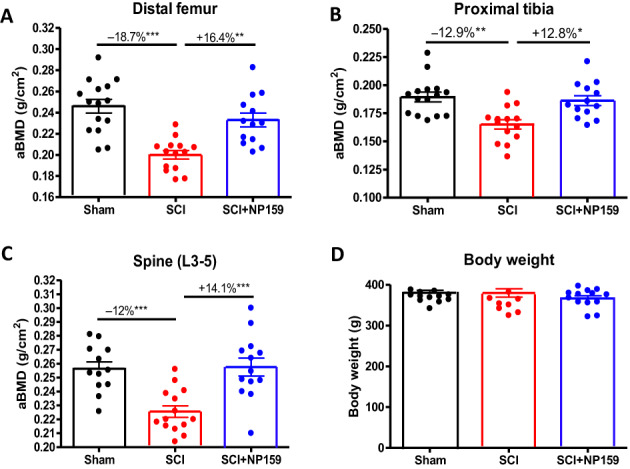
Areal BMD for indicated sites determined by analysis of images acquired by dual‐energy X‐ray absorptiometry scanning and body weight measurement. (A) Distal femur, (B) proximal tibia, (C) spine (L3–5), and (D) body weights. Sham‐operated animals were used as controls. Data are expressed as mean ± SEM. *N* = 13 to 15 per group. Significance of differences was determined using one‐way ANOVA with Newman–Keuls test post hoc. **p < 0.01 and ***p < 0.001 versus indicated group.

To test our hypothesis, we conducted preclinical animal studies to determine how administration of NP159 altered bone loss in male rats that had a complete spinal cord transection at the fourth thoracic vertebra. In these studies, 4‐month‐old rats were administered NP159 (20 mg/kg biweekly) or vehicle beginning immediately after SCI and until the animals were euthanized 56 days after SCI. At the end of the study, there were no significant differences in body weights among sham, SCI, and SCI + NP159 animal groups, either before performing surgery or at time of sacrifice (Fig. 1F). Eight weeks after SCI, the BMD of the distal femur, proximal tibia, and spine (L3‐5) were diminished by −18.7%, −12.9%, and −12.0%, respectively. Of note, NP159 treatment completely prevented the loss of BMD at each of these skeletal sites after acute SCI (Fig. 1A–C).

**Fig. 2:**
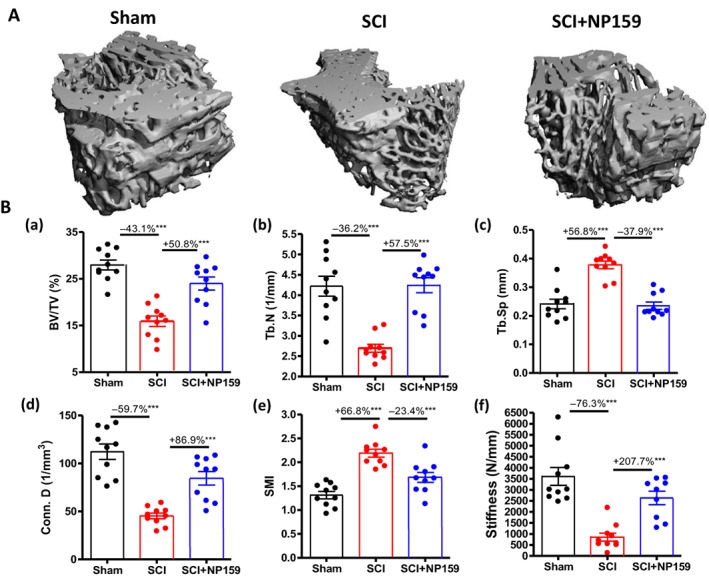
Effect of NP159 on trabecular architecture of distal femur as assessed by μCT. (A) Representative 3D images of trabecular microarchitecture. Measurements are shown for (B) (a) trabecular bone volume over total volume (BV/TV); (b) trabecular number (Tb.N, mm^−1^); (c) trabecular separation (Tb.Sp, μm); (d) connectivity density (Conn.D, mm^−3^); (e) structure model index (SMI, ranges from 0 to 3 with 0 = platelike and 3 = rodlike); (f) trabecular thickness (Tb.Th, μm); and (g) bone stiffness (N/mm) by finite element analysis, respectively. Data are expressed as mean ± SEM. *N* = 10 per group. Significance of differences was determined using one‐way ANOVA with a Newman–Keuls test post hoc. *p < 0.05, **p < 0.01 and ***p < 0.001 versus indicated group.

Bone architecture was examined by high‐resolution μCT to assess changes in trabecular bone of the distal femoral metaphysis (Fig. 2). After SCI, trabecular bone volume (BV/TV%) at this site was reduced by nearly −43.1% (*p* < 0.001, Fig. 2B[a]) due largely to decreased trabecular number (Tb.N) (−36.2%, *p* < 0.001, Fig. 2B[b]) with an increase in trabecular space (Tb.Sp) (+56.8%, *p* < 0.001, Fig. 2B[c]). Trabecular connectivity density (Conn.D) was greatly reduced (−59.7%, *p* < 0.001, Fig. 2B[d]), associated with transformation from platelike to rodlike structures (structure model index [SMI], +66.8%, *p* < 0.001, Fig. 2B[e]). Administration of the NP159 almost completely prevented declines in BV/TV% (+50.8%, *p* < 0.001, Fig. 2B[a]) and Conn.D (+86.9%, *p* < 0.001, Fig. 2B[d]), primarily by increasing Tb.N (+57.5%, *p* < 0.001, Fig. 2B[b]) and preserving Tb.Sp (−37.9%, *p* < 0.001, Fig. 2B[c]). Of note, we previously demonstrated that, in contrast to NP159, antagonism by sclerostin antibody failed to prevent the loss of Conn.D and Tb.N after acute SCI, despite its nearly full protection against the loss of BMD and trabecular BV/TV%.^[^ ^10^ ^]^ μFEA revealed that SCI reduced bone stiffness by −76.3% at the distal femur (*p* < 0.001), and the decline in bone mechanic property was dramatically blocked by NP159 administration (+207.7%, *p* < 0.001, Fig. 2B[f]).

Cortical bone structure and strength at the femur midshaft was also examined by high‐resolution μCT and μCT‐based μFEA. Compared with those from the sham group, bones at 56 days after SCI became thinner with a reduction in cortical thickness (Ct.Th; −7.0%, *p* < 0.05; Fig. S1A), accompanied by a significant reduction in bone stiffness (−8.8%, *p* < 0.01; Fig. S1B). The decline in bone mechanical property was significantly blocked by NP159 administration (+6.2%, *p* < 0.01, Fig. S1B).

### Siglec‐15 antibody greatly inhibited bone resorption while maintaining bone formation

**Fig. 3:**
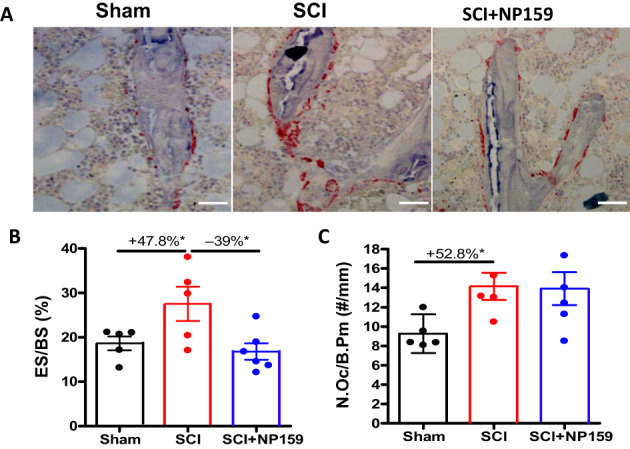
Effects of NP159 on bone resorption of trabecular bone at distal femur. (A) Representative sections of trabecular bone from femoral metaphysis immunostained for TRACP (magnification ×20). The reddish areas of TRACP staining on trabecular surfaces represent osteoclasts. These were all measured/taken in the area 1 mm underneath the growth plate. (B–C) Parameters of trabecular bone resorption by histomorphometric quantification. (B) Eroded surface/bone surface (ES/BS) and (C) osteoclast number over bone perimeter (N.Oc/B. Pm). Scale bar = 50 mm. Data are expressed as mean ± SEM. *N* = 6 or 7 per group. Significance of differences was determined using one‐way ANOVA with Newman–Keuls test post hoc. *p < 0.05 versus the indicated group.

Static histomorphometric analysis was conducted to evaluate bone resorption. Sections of trabecular bone from the femur were immunostained for TRACP. SCI resulted in increased eroded surface/bone surface (ES/BS; +47.8%, *p* < 0.05, Fig 3B) and osteoclast number/bone perimeter (N.Oc/B.Pm; +52.8%, *p* < 0.05, Fig 3C). NP159 treatment in SCI rats greatly reduced ES/BS (−39%, *p* < 0.05, Fig 3B) compared to controls, suggesting inhibition of bone resorption. Because TRACP staining can detect both mononuclear (immature) and multinuclear osteoclasts (MUC, mature), no obvious changes in N.Oc/B.Pm (Fig 3C) in SCI + NP159 rats relative to the SCI group suggest that NP159 mainly reduced the number of functional MUC for resorbing bone but did not alter the number of mononuclear preosteoclasts (Fig. 3), an observation that is consistent with previous studies.^[^ ^32^, ^57^ ^]^

**Fig. 4:**
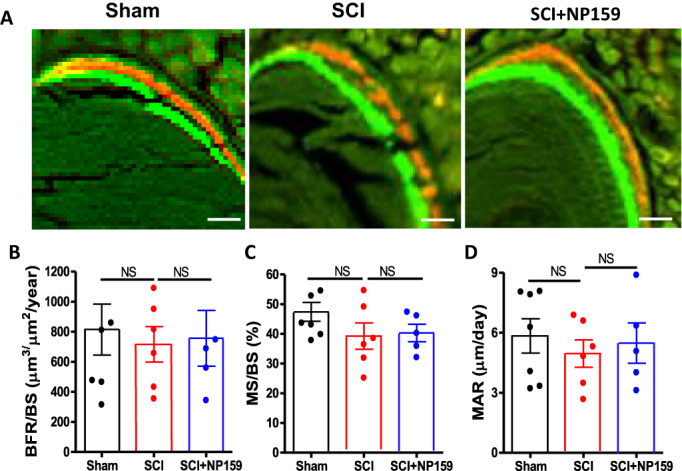
Effects of NP159 on bone formation of trabecular bone at distal femur. (A) Representative 6‐μm‐thick bone specimen showed double‐labeling of calcein green and xylenol orange under fluorescence microscopy (magnification ×20). Measurement is shown for (B) BFR/BS (mm^3^/mm^2^/year), (C) MS/BS (%), and (D) MAR (mm/day). These were all measured/taken in the area 1 mm underneath the growth plate. Scale bar = 50 mm. Data are expressed as mean ± SEM. *N* = 5 to 7 per group. Significance of differences was determined using one‐way ANOVA with Newman–Keuls test post hoc. NS, no significance. BFR/BS, bone formation rate/bone surface; MS/BS, mineralizing surface/bone surface; MAR, mineral apposition rate.

Dynamic histomorphometric analysis revealed no significant changes in mineralizing surface/bone surface (MS/BS), mineral apposition rate (MAR), and bone formation rate/bone surface (BFR/BS) in the NP159‐treated group compared to the SCI control group (Fig. 4). These results indicate that NP159 maintains bone formation while inhibiting bone resorption, in contrast to other antiresorptive drugs (e.g., BPs and DMAB) that suppress bone formation.^[^ ^58^ ^]^

**Fig. 5:**
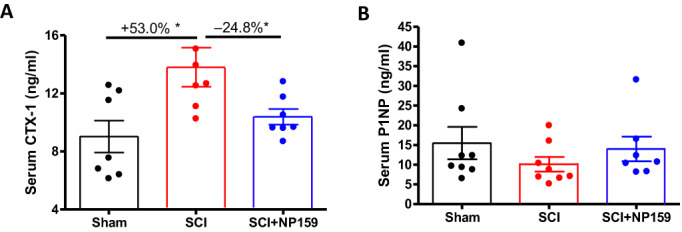
Effects of NP159 on levels of serum biomarkers of bone resorption and formation. ELISA tests for (A) CTX and (B) P1NP. Data are expressed as mean ± SEM, *N* = 10 to 13 per group. Significance of differences was determined using one‐way ANOVA with a Newman–Keuls test post hoc. *p < 0.05, **p < 0.01, ***p < 0.001.

ELISA assays were performed to examine the serum levels of bone biomarkers for resorption, CTX, formation, P1NP, and osteocalcin. Consistent with previous findings, CTX levels were increased after SCI (+10.3%, *p* < 0.05, Fig. 5A).^[^ ^34^, ^41^ ^]^ Importantly, we found NP159 significantly decreased serum CTX levels (−13.3%, *p* < 0.05, Fig. 5A), suggesting inhibition of bone resorption. Following acute SCI, serum P1NP level is decreased by −34.7%. NP159 treatment increased the concentration of P1NP in blood, although this change did not reach statistical significance (Fig. 5B). We observed no change in the level of osteocalcin with NP159 treatment following SCI (Fig S2).

Thus, the blood biomarker‐based readouts of both bone formation and resorption seen following treatment of SCI rats with NP159, consistent with changes in bone seen with the histomorphometric analysis of bone formation and resorption. Collectively these data support a mechanism for the Siglec‐15 Ab, NP159, where NP159 inhibits bone resorption while maintaining bone formation after acute SCI.

### Siglec‐15 antibody inhibited osteoclastogenesis

**Fig. 6:**
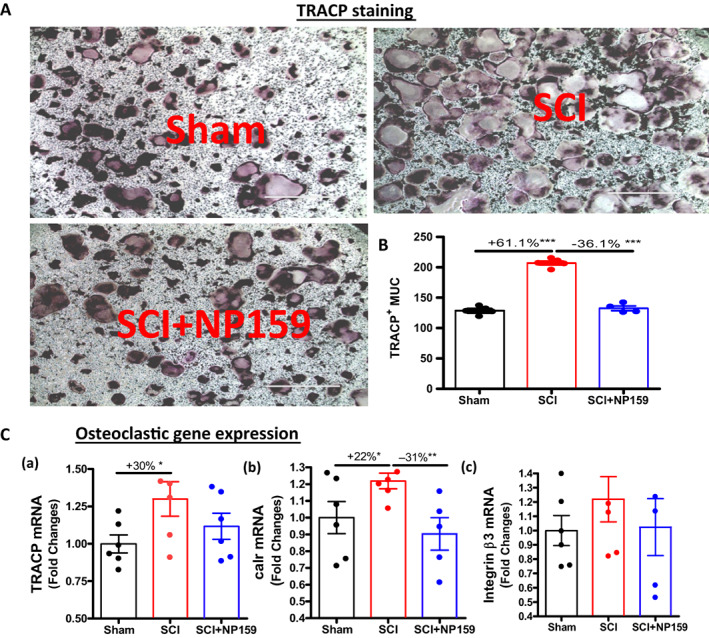
NP159 inhibited the osteoclastogenesis of bone marrow stem cells. (A) Shown are representative images of cultures of hematopoietic progenitors cultured with M‐CSF and RANKL then stained for TRACP. (B) Cell counts for TRACP^+^ multinucleated cells are shown. (C) Changes in gene expression of bone resorption markers in cultured osteoclasts were determined by qPCR: (a) TRACP, (b) calreticulin (Calr), and (c) integrin β3. Scale bar = 1,000 mm. *N* = 4 or 5 per group. Significance of differences was determined using one‐way ANOVA with a Newman–Keuls test post hoc. *p < 0.05, **p < 0.01, ***p < 0.001 versus indicated group.

Bone marrow cells can be separated into hematopoietic precursor cell (HPC) and mesenchymal precursor cell (MSC) populations, which can then be differentiated into multinucleated TRACP^+^ osteoclasts capable of resorbing bone and alkaline phosphatase (ALP)‐positive osteoblasts that form and mineralize osteoid, respectively. Following immobilization, the potential of MSCs to undergo osteoclastogenic differentiation is increased.^[^ ^59^, ^60^ ^]^ At 21 or 56 days after SCI, marrow HPCs were assessed for osteoclastogenic differentiation activity and showed enhanced differentiation into osteoclasts.^[^ ^10^, ^40^, ^61^ ^]^ In this study, we performed osteoclastogenesis and osteoblastogenesis assays to examine the effects of NP159 on the ability of marrow cells to differentiate in cell cultures into osteoclasts or osteoblasts. Consistent with our previous findings,^[^ ^10^, ^34^, ^47^ ^]^ after SCI both the number of TRACP^+^ MUC (+61.1%, *p* < 0.001) and the expression of osteoclast marker genes TRACP and calcitonin receptor (Calr) were significantly increased when measured ex vivo. Of note, NP159 markedly decreased TRACP^+^ MUC (−36.1%, *p* < 0.001) and TRACP and Calr mRNAs (Fig. 6), indicating the reduction of osteoclast maturation and osteoclastogenesis in vivo.

### Siglec‐15 antibody enhanced osteoblastogenesis

**Fig. 7:**
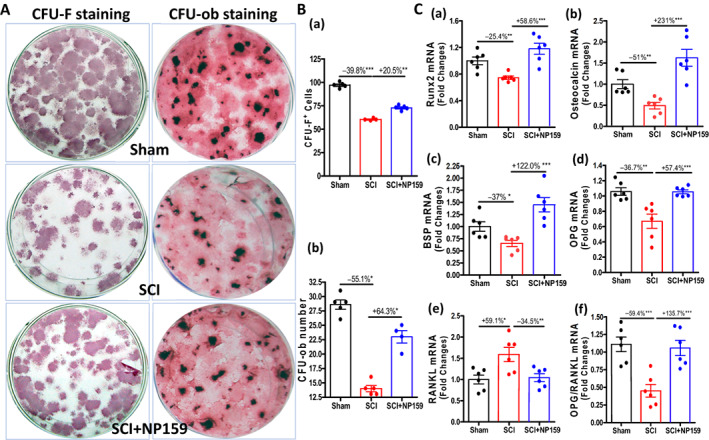
NP159 enhanced the osteoblastogenesis of bone marrow stem cells. (A) Representative images showing alkaline phosphatase staining (CFU‐F) and Von Kossa staining (CFU‐ob) of cultures of marrow stromal cells. (B) Cell counts of alkaline phosphatase‐positive cells and formed bone nodules. (C) Changes in gene expression in osteoblasts developed by primary culture of bone marrow stromal cells. mRNA levels were determined by real‐time PCR: (a) Runx2, (b) osteocalcin, (c) BSP, (d) OPG, (e) RANKL, and (f) OPG/RANKL ratio. Data are expressed as mean ± SEM. *N* = 4 to 5 per group. Significance of differences was determined using one‐way ANOVA with a Newman–Keuls test post hoc. *p < 0.05, **p < 0.01, and ***p < 0.001 versus indicated group.

Consistent with our previous findings,^[^ ^10^, ^33^, ^35^ ^]^ the decrease in the number of osteoblast‐forming cells (examined by CFU‐F staining), the number of colonies producing mineralized bone matrix (examined by von Kossa staining, CFU‐ob), and the levels for transcripts encoding the osteoblast differentiation markers Runx2, osteocalcin (OCN), and bone sialoprotein (BSP) were observed in rats following SCI (Fig. 7A,B[a,b]). Notably, comparison of NP159‐treated SCI rats to controls revealed a significant increase in the numbers of CFU‐F^+^ staining (+20.5%, *p* < 0.01) or mineralized nodule (CFU‐ob) cells (+64.3%, *p* < 0.01) (Fig. 7B[a,b]). In ex vivo cultures of osteoblasts derived from MSCs, mRNA levels of Runx2, OCN, and BSP were significantly increased in the SCI + NP159 group compared to the SCI group (Fig. 7C[a–c]). Interestingly, levels of mRNA for osteoprotegerin (OPG) were reduced by about −36.7% (*p* < 0.01) for the SCI group compared with the sham group, associated with +59.1% elevations (*p* < 0.05) in mRNA levels for RANKL. Treatment with NP159 significantly increased OPG mRNA and reduced mRNA levels for RANKL, resulting in a 135.7% increase (*p* < 0.001) in the OPG/RANKL ratio (Fig. 7C[d–f]). Collectively, our data indicate that NP159 induced osteoblastogenesis while suppressing osteoclast maturation and osteoclastogenesis (Fig. 6).

## Discussion

This study constitutes the first investigation demonstrating the efficacy of a novel therapeutic, Siglec‐15 Ab, that reduces bone resorption after acute SCI while maintaining capacity for bone formation as well. Hence, NP159 stands out as a unique therapeutic agent in bone disease, including disuse osteoporosis, in that it has antiresorptive activity while preserving bone formation function for a net anabolic outcome. The bone loss after SCI is particularly rapid, progressive, and severe. The fragility of regions of the skeleton in individuals with immobilizing neurological conditions places them at increased risk for fractures. This results in hospitalization stays, increased costs for medical care, and decreased overall quality of life. Despite the pressing nature of this problem, to date, there is no practical approach to safely and efficaciously treat bone loss to prevent fractures in individuals with chronic SCI. This study addresses this critical need for an effective and safe therapeutic approach for paralyzed individuals with severe bone fragility. In this study, we have demonstrated that a novel Siglec‐15 Ab, NP159, almost fully prevents the SCI‐induced loss of bone mass and preserves trabecular microarchitecture in a rat model of acute motor‐complete spinal cord transection. NP159 decreases osteoclast maturation and bone resorption while increasing osteoblastogenesis to maintain bone formation in SCI. This study is the first to demonstrate that a Siglec‐15 Ab is effective at mitigating bone loss in immobilizing conditions, which includes not only after SCI but should also extend to other disuse conditions that result in severe osteoporosis. Blockade of Siglec‐15 to preserve skeletal health after acute SCI‐induced immobilization is original and innovative. Whether this agent can reverse the bone loss in chronic SCI has yet to be tested. If efficacious in a preclinical model of chronic SCI, this agent may be quickly deployed in a clinical trial. If skeletal integrity can be improved in persons with chronic SCI, this approach would hold the promise to increase the number of individuals who would have been denied access, but may become eligible, for rehabilitation strategies (e.g., exoskeletal‐assisted walking, spinal cord stimulation) or other modalities for gait to allow greater functional independence.

There are limitations to the current pharmacological interventions in SCI‐induced bone loss. Two broad classifications of agents are currently approved by the US Food and Drug Administration (FDA) for the treatment of osteoporosis: antiresorptive agents and anabolic agents. As one class of antiresorptive agents, BPs reduce osteoclast viability, number, and bone‐resorbing activity. BPs have been found to increase bone mass and reduce fractures in postmenopausal women and stroke survivors.^[^ ^62^, ^63^ ^]^ These agents are now appreciated to have an additional mechanism of action by regulating the activity of connexin‐43 hemichannels on osteocytes and osteoblasts.^[^ ^64^ ^]^ Several trials have evaluated the effects of BP administration on bone loss after acute SCI.^[^ ^65^, ^66^, ^67^ ^]^ Treatment with BPs, at best, slows bone loss at some sites after acute motor‐complete SCI but does not spare bone at the most clinically relevant sublesional locations. Bauman et al. reported that subjects with motor‐complete SCI who were administered two different BP drugs, pamidronate^[^ ^65^ ^]^ or zoledronic acid,^[^ ^68^ ^]^ did not preserve BMD at the knee (the distal femur and proximal tibia), the skeletal site at highest risk of fracture. The use of BPs is also associated with a number of adverse side effects that are similar to those seen with another antiresorptive agent, DMAB.

DMAB, a human monoclonal Ab to RANKL, represents an immunopharmacological approach to the treatment of osteoporosis that has been approved by the FDA. The mechanism of action of DMAB, which is to prevent the recruitment and the development of osteoclasts, is distinctly different from that of BPs. DMAB has been demonstrated to be an effective agent in postmenopausal osteoporosis in a number of clinical trials, including the Fracture Reduction Evaluation of DMAB in Osteoporosis Every 6 Months (FREEDOM) Trial.^[^ ^69^ ^]^ Histomorphometric findings suggest that the effects of DMAB on bone remodeling are more potent than those with BPs.^[^ ^70^, ^71^ ^]^ Emerging findings from our clinical investigators have shown that DMAB greatly reduced bone loss after subacute SCI.^[^ ^11^ ^]^ While these findings are quite promising, DMAB also potently suppresses osteoblast activity and bone formation when it inhibits the activity of osteoclasts,^[^ ^72^ ^]^ similar to that of BPs. The coupled inhibition of bone resorption and formation is believed to be responsible for the rare, but devastating, side effects of DMAB and BPs reported in the general population (e.g., osteonecrosis of the jaw and atypical femoral fractures).^[^ ^71^, ^73^, ^74^ ^]^ A controversy also exists concerning the efficacy of BPs in the treatment of bone disease in those with chronic SCI largely due to BP‐mediated inhibition of osteoblast activity and bone formation while inhibiting osteoclast activity and bone resorption.^[^ ^65^, ^66^, ^75^ ^]^ In addition, able‐bodied patients prescribed DMAB have also reported trouble breathing, backache, and an ill‐defined pain in muscle and/or bone.^[^ ^76^ ^]^ Recent evidence suggests that the cessation of DMAB might be accompanied by a period of accelerated bone resorption and increased fracture risk, perhaps through a mechanism by which osteomorph (“fissioned” from mature osteoclasts) can refuse and recycle to form functional osteoclasts in a RANKL/RANK/OPG signaling‐dependent manner.^[^ ^77^ ^]^

With regard to bone anabolic agents, currently there are three FDA‐approved agents with bone anabolic activity: teriparatide, abaloparatide, and ROMO. Teriparatide is a peptide containing amino acid residues 1–34 of PTH that is effective at reducing risk of fractures in postmenopausal osteoporosis.^[^ ^78^, ^79^ ^]^ However, two recent studies did not observe significant changes in BMD of the lower extremities of individuals with SCI after 6 months of teriparatide coupled with mechanical loading or vibration,^[^ ^80^, ^81^ ^]^ suggesting an uncertain role of teriparatide in the treatment of bone loss after SCI. Currently, the exact reason that these treatment approaches applied after SCI are ineffective remains unknown. However, it would be reasonable to speculate that the ineffectiveness might be associated with the multiple pathogenic circumstances responsible for bone loss, which include anticipated neurological, inflammatory, and hormonal factors, in addition to extreme, protracted immobilization. As a consequence, the rate and extent of bone loss after SCI is substantially greater than those of other pathological conditions predisposing to osteoporosis, such as prolonged bedrest, microgravity during spaceflight, or postmenopausal osteoporosis.^[^ ^14^ ^]^ Another major concern is that teriparatide and abaloparatide (PTH‐related protein analog) treatment is limited to a 2‐year period in patients because of the increased potential risk of developing osteosarcoma with a longer duration of therapy.^[^ ^82^, ^83^ ^]^

Monoclonal human Ab against sclerostin, ROMO, that targets the Wnt signaling pathway was recently approved as a new and potent anabolic agent for the treatment of women with postmenopausal osteoporosis at high risk of fracture. In women with postmenopausal osteoporosis, ROMO can increase BMD and bone formation while decreasing bone resorption.^[^ ^84^ ^]^ ROMO significantly reduced the incidence of new vertebral fractures in the Fracture Study in Postmenopausal Women with Osteoporosis (FRAME), but the drug failed to meet the secondary objective of reducing the incidence of nonvertebral fractures.^[^ ^85^ ^]^ However, ROMO could significantly reduce nonvertebral fractures by 19% in a separate study.^[^ ^86^ ^]^ Importantly, the latter study also reported that patients on ROMO had a slightly higher risk of serious cardiovascular events than the comparator drug (alendronate),^[^ ^86^ ^]^ a risk that is still under debate.^[^ ^87^ ^]^ Recent preclinical work from our group and others strongly supports sclerostin Ab as an attractive agent to decrease bone loss after acute SCI,^[^ ^10^, ^40^, ^88^ ^]^ as well as to reverse bone loss in chronic SCI.^[^ ^35^ ^]^ However, the use of the drug has been limited by the FDA to women and only for 1 year as the drug carries a black box warning from the FDA cautioning its use in patients at higher risk for cardiovascular disease and stroke, two risk factors that are elevated in patients with SCI.^[^ ^89^ ^]^ When considering the uncertain efficacy of ROMO in reducing nonvertebral fractures, along with the possibility of increased cardiovascular side effects, FDA approval for use only in women, and relatively short period of safe therapeutic administration, the need to develop an alternative therapeutic approach is very clear. The most frequent fracture in patients with SCI occurs at the distal femur and proximal tibia (e.g., the knee region), which is a nonvertebral site. SCI is an ongoing, life‐long condition, and, as such, bone loss that follows SCI is a process that continues for decades.^[^ ^14^, ^16^ ^]^ Thus, even if ROMO proves to be efficacious, this agent would not fully meet the need for the treatment of bone loss in those with chronic SCI. The availability of an agent that will reduce nonvertebral fractures and be safely administered for a longer period of time would be of high clinical relevance to improve long‐term bone health in patients with chronic SCI.

Considering the various limitations associated with the current pharmacological interventions in SCI‐induced bone loss, we believe that NP159 offers a novel, potent, and potentially safer drug for use in improving skeletal integrity after SCI. NP159 can inhibit osteoclast maturation and activity while preserving osteoblast function in SCI, a feature distinct from the current antiresorptive agents (e.g., RANKL Ab and BPs) that inhibit the activity of both osteoclasts and osteoblasts.^[^ ^65^, ^66^, ^75^ ^]^ As such, Siglec‐15 Ab is capable of offering better efficacy than that of BPs and DMAB in SCI‐induced bone loss because the latter agents result in the suppression of bone formation, an effect that may have resulted in questionable efficacy of BPs in treating bone disease in those with SCI.^[^ ^65^, ^66^, ^75^ ^]^ We acknowledge the observation that a net increase in BMD is progressive with DMAB treatment, which suggests a greater suppression of bone resorption than bone formation.^[^ ^69^ ^]^ However, the bone turnover and the healing of microfractures may be impeded by long‐term treatment with a potent antiresorptive agent, predisposing to atypical femoral fractures.^[^ ^71^, ^74^ ^]^ The potentially superior safety profile of Siglec‐15 Ab may allow it to be safely used for more extended periods of time to treat bone loss in those with chronic SCI; this is in contrast to the commercially available bone anabolic agents (teriparatide, abaloparatide, and ROMO), which are limited in treatment length of 1–2 years due to safety concerns, or the other antiresorptive agents that may cause osteonecrosis of the jaw and atypical femoral fractures or other adverse effects.

Importantly, the present work strongly suggests that inactivating Siglec‐15 by NP159 has a favorable influence on the differentiation potential of bone marrow progenitors by inhibiting osteoclastogenesis while promoting osteoblastic differentiation. We demonstrated that NP159 administration led to the upregulation of OPG and the OPG/RANKL ratio in cultured osteoblasts (Fig. 7[d–f]), thereby resulting in diminishing the elevated osteoclast population (TRACP^+^ multinucleated cells) after SCI in bone marrow hematopoietic stem cells (Fig. 6). Such an NP159‐mediated indirect effect is in addition to the known direct effect of Siglec‐15 inhibition to block Siglec‐15‐DAP12 complex formation and inactivate Akt signaling pathway that can prevent osteoclast fusion and maturation.^[^ ^25^, ^27^, ^90^ ^]^ In addition, we found that NP159 increased the number of CFU and calcified nodules in ex vivo cultured osteoblasts. This enhanced osteoblastogenesis results in maintaining bone formation (Figs. 4 and 5). The elevated osteogenesis induced by Siglec‐15 antagonism in this study is consistent with previous findings showing an increased number of osteocalcin‐positive osteoblasts, an enlarged osteoid‐covered bone surface, and greater trabecular bone volume in the Siglec‐15 knockout mice compared to wild‐type mice.^[^ ^32^ ^]^ In the present study, however, a discrepancy exists with regard to bone formation parameters between the histomorphometric evidence in bone tissue sections and the cellular observations in cultured bone marrow cells: an increased osteoblastogenesis is seen for the latter, while evidence of bone formation is not seen for the former with NP159 treatment after SCI. It is not clear what explains the observed difference in bone formation on bone surfaces versus the potential of bone marrow progenitors to undergo osteoblastogenic differentiation. In response to Siglec‐15 inhibition, it seems that while bone marrow stem cells are primed to differentiate into osteoblastic cells, some critical elements necessary for them to do so are not present. It is also important to note that at the earlier stage of SCI, bone formation rate did not change at 2 months after injury in this study and at up to 6 months in a previous study^[^ ^91^ ^]^; thus, one might not expect NP159 to increase bone formation despite the fact that NP159 has the potential to promote osteoblastogenesis in bone MSCs to support bone formation. It remains possible that NP159 can promote bone formation rate at the later stage of SCI when bone formation is greatly compromised.^[^ ^14^, ^18^ ^]^ In addition, it would also be of interest in a future study to investigate whether, in the conditions of SCI‐induced immobilization, Siglec‐15 Ab can act by blocking the Siglec‐15‐DAP12 complex for inactivating Akt signaling to inhibit osteoclast fusion and maturation,^[^ ^27^, ^90^ ^]^ promoting the production of PDGF‐BB from preosteoclasts for recruitment of more osteoblasts, or other mechanisms,^[^ ^32^ ^]^ thereby exerting its antiresorptive roles while sparing or even promoting bone formation.

It is of great interest to know whether the benefits to bone after acute SCI in rats can translate to more sustained protection against significant bone loss after chronic SCI. Future work can be conducted to investigate the effects of Siglec‐15 Ab or the combination of Siglec‐15 Ab with other pharmacological or nonpharmacological approaches to offer therapeutic benefit on bone after a more extended period of time following SCI, when a number of pathological changes, including those of prolonged immobilization, severe neurological impairments, and systemic hormonal and metabolic dysfunctions, are more fully developed and when robust bone loss at the level below spinal cord lesion has already happened, as is the case for most patients with chronic SCI who have longstanding paralysis. Current knowledge and our prior works support the hypothesis that Siglec‐15 Ab has a high likelihood of improving bone health in chronic SCI. It has been shown that gains in bone mass by functional electrical stimulation may occur even years after SCI.^[^ ^92^ ^]^ Microgravity in spaceflight results in significant bone loss, but 1 year of gravitational loading in most astronauts after returning to earth generally leads to significant reversal of these skeletal changes.^[^ ^93^ ^]^ Our compelling data demonstrate that Siglec‐15 Ab almost fully prevents bone loss and preserves trabecular microarchitecture in acute SCI via its potent antiresorptive activity (inhibiting osteoclast maturation for suppressing osteoclast activity) coupled with anabolic action (promoting osteoblastogenesis to maintain or even increase bone formation). The unique feature of Siglec‐15 action in maintaining the capacity for bone formation while inhibiting bone resorption should allow for new bone accrual to restore bone mass and, possibly, structure. This should lead to improved skeletal integrity after marked bone loss in chronic SCI. Another limitation of this work was that only male rats were studied. It thus remains of interest and relevance to investigate whether the beneficial effects can be extended to females.

We also found that, similar to the antiresorptive and anabolic effects of Siglec‐15 Ab in acute SCI, sclerostin Ab administration demonstrated anabolic action (enhancing bone formation) through activation of Wnt signaling and protected against bone deterioration after acute SCI, coupled with moderate antiresorptive activity.^[^ ^10^ ^]^ However, it is important to note that a key differentiator between the two treatments is that the favorable changes in Tb.N and Conn.D were observed by administration of NP159 (Fig. 1B[b,d] in the present study) but not by sclerostin Ab (Fig. 1F,G in our previous report^[^ ^10^ ^]^). This suggests that antagonizing Siglec‐15‐mediated activity in bone leads to a better outcome in improving microarchitecture than sclerostin antagonism does. Furthermore, we reported that sclerostin Ab‐mediated skeletal benefits (e.g., complete reversal of marked sublesional bone loss and normalization of bone strength) can be extended to the condition of chronic SCI when applied 3 months after SCI,^[^ ^35^ ^]^ suggesting that bone restoration remains effective even after a relatively long interval after SCI and that it may also be possible to reverse loss of bone mass and strength after chronic SCI. Based on these considerations, it is highly probable that Siglec‐15 Ab administration can greatly restore the marked sublesional bone loss in chronic SCI in a future preclinical study, if not to a greater extent, at least to a degree similar to that observed for that of sclerostin Ab administration.^[^ ^35^ ^]^

Because the main mechanism of action of NP159 is to inhibit osteoclast fusion, the number of osteoclast precursors would be unchanged. Substantial “rebound” bone loss has been reported following discontinuation of DMAB treatment,^[^ ^94^ ^]^ and this may also be a clinical concern when discontinuing NP159 treatment, which will require future work to determine the magnitude of bone loss that would likely occur when Siglec‐15 antagonism is discontinued. Of note, another form of Siglec‐15 Ab (DS‐1501a) has proven to be potent and have an excellent safety profile in the clinical trial to date.^[^ ^31^ ^]^ We note that in the present study, NP159 treatment did not cause animal death, weight loss, or other adverse effects in SCI rats. Preliminary toxicology studies suggest that NP159 treatment in rodents is safe. Analysis of tissues tested (thymus, heart, lung, liver, spleen, kidney, inguinal lymph node) showed no major histopathology finding after five doses up to 30 mg/kg biweekly for 2 months in mice (NextCure, personal communication, May 2023). In addition, no adverse effects were observed after seven doses at 10 mg/kg or in rats treated. If NP159 can demonstrate a safety profile in clinical trial similar to that of DS‐1501a, Siglec‐15 Ab can be considered a potentially novel agent that could be administered for longer periods of time than other anti‐osteoporotic agents that are currently available on the market.

In conclusion, when started immediately after SCI and continued for 8 weeks in a rodent model of motor‐complete SCI, administration of Siglec‐15‐inactivating antibody was demonstrated to lead to a pronounced increase in bone mass and a preservation of trabecular microarchitecture in skeletons below the level of spinal cord transection. Mechanisms underlying the antiresorptive and anabolic activities of Siglec‐15 Ab are largely attributed to its suppression of osteoclast maturation and bone resorption while increasing osteoblastogenesis to maintain bone formation. Our findings establish Siglec‐15 Ab as a novel antiresorptive, but also as a uniquely bone formation‐sparing agent in animals with motor‐complete SCI. Siglec‐15 Ab has been demonstrated to have potent efficacy in reducing marked bone loss, as well as having a fine safety profile, as shown in prior human studies. Because administration of Siglec‐15 Ab exhibits potent bone anabolic actions after paralysis from SCI, there is reason to assume that it would also have efficacy in the treatment of other conditions associated with extreme immobilization. Our discoveries have significant clinical implications because skeletal deterioration in individuals with motor‐complete SCI is rapid, progressive, severe, and refractory to the pharmacologic approaches available to date, except that of DMAB.^[^ ^13^, ^14^, ^68^ ^]^ Thus, our findings from this study strongly indicate that antibody‐mediated blockade of Siglec‐15 can serve as a promising novel therapeutic option to preserve skeletal mass and integrity after acute SCI, as well as for the treatment of other conditions of severe osteoporosis associated with chronic immobilization and disuse (e.g., prolonged bedrest and spaceflight), neurologic etiologies (e.g., stroke, Parkinson's disease, amyotrophic lateral sclerosis, and multiple sclerosis), or activity‐limiting rheumatological diseases.

## Author Contributions

**Yuanzhen Peng:** Data curation; formal analysis; investigation; validation. **Solomon Langermann:** Funding acquisition; resources; validation; writing – review and editing. **Priyanka Kothari:** Resources; writing – review and editing. **Linda Liu:** Project administration; resources; writing – review and editing. **Wei Zhao:** Data curation. **Yizhong Hu:** Data curation. **Zihao Chen:** Data curation. **Mariana Moraes de Lima Perini:** Data curation; methodology. **Jiliang Li:** Data curation; methodology. **Jay J. Cao:** Data curation; methodology. **X. Edward Guo:** Data curation; resources. **Lieping Chen:** Data curation; methodology; resources. **William A. Bauman:** Resources; writing – review and editing. **Weiping Qin:** Conceptualization; data curation; formal analysis; funding acquisition; investigation; methodology; project administration; resources; supervision; validation; writing – original draft; writing – review and editing.

## Disclosures

YP, WZ, YH, ZC, JL, JC, XEG, and WB have nothing to disclose. SL, PK, LL, and LC are current or former employees and shareholders of NextCure, Inc. NextCure, Inc. and WQ have jointly filed a patent application for intellectual protection. LL's current address: Zai Laboratory, USA.

### Peer Review

The peer review history for this article is available at https://www.webofscience.com/api/gateway/wos/peer-review/10.1002/jbm4.10825.

## Supporting information


**Data S1.** supporting Information.

## Data Availability

The data that support the findings of this study are available from the corresponding author upon reasonable request.
