# Supplementary material for: Anti‐Siglec‐15 Antibody Prevents Marked Bone Loss after Acute Spinal Cord Injury‐Induced Immobilization in Rats
Source: JBMR Plus. 2023 Sep 27;7(12):e10825. doi: 10.1002/jbm4.10825 (PMC10731123; doi:10.1002/jbm4.10825)
Supplement: Supplementary file 1 — Data S1. supporting Information. [file JBM4-7-e10825-s001.docx]

**Supplementary Materials and Methods**

*1. Animal Surgery*

Complete spinal cord transection in rodents were conducted as previously described [1-8]. Briefly, 4-months old Wistar male rats (Charles River) were anesthetized by inhalation of isofluorane (3-5%) and hair was removed with a clipper. Skin over the back was cleaned with betadine and isopropyl alcohol. After making a midline incision the spinal cord at the site of transection (at interspace between the 3^rd^ and 4^th^ vertebral bodies) was visualized by removing the vertebral process above and below the level of transection with a bone rongeur, and the spinal cord was transected with microscissors. The space between transected ends of the spinal cord was filled with surgical sponge and the wound was closed in 2 layers with suture. Bladder was expressed 3 times daily until automaticity developed, then as needed. Baytril wasadministered for the first 3 to 5 days postoperatively then as indicated for cloudy or bloody urine or for overt wound infection. Sham-transected animals received an identical surgery, including a laminectomy, except that the spinal cord was not cut.

*2. Tissue Collection*

Blood was collected by cardiac puncture. The leg was removed using sterile technique and careful dissection to free the head of the femur from the pelvis. To preserve bone for micro-CT and histomorphometry studies, the left leg was removed and placed into tubes containing 4% PFA overnight, after which the PFA will be replaced with 70% ethanol for storage. For cell culture studies, the right leg was placed into sterile tubes containing ice-cold Minimum Essential Alpha Medium, then kept at 4˚C until collection of marrow cells.

*3. Dual Energy X-ray Absorptiometry Analysis of BMD*

Areal BMD measurements were performed on all the collected bone samples by using a small animal dual energy X-ray absorptiometer (DXA) (Lunar Piximus, Fitchburg, WI, USA) as described previously [1, 2, 9, 10]. The instrument was calibrated using a phantom following the manufacturer's recommendations on each day of use. Hindlimbs were positioned on the densitometer platform with the knee flexed at an angle of 135°, and DXA images were acquired with Lunar Piximus software. The metaphyses of the distal femur and proximal tibia were selected as ROI. The precision for BMD measurements (coefficients of variation) is approximately 1.5% for the ROI.

*4. MicroCT Analysis of Trabecular Architecture*

To evaluate trabecular architecture of the distal femur, microCT was performed on fixed bones, as described previously using a Scanco μCT scanner and a 16-μm voxel size [2, 10, 11]. The femur was placed in a holder and scanned non-destructively by using a Scanco µCT scanner (µCT-40; Scanco Medical AG, Bassersdorf, Switzerland) at 16 µm isotropic voxel size (the highest resolution) with X-ray source power of 55 kV and 145 µA and integration time of 300 milliseconds. The trabecular microstructure of the distal femur was evaluated. The scanned grey-scale images were processed by using a low-pass Gaussian filter (sigma = 0.8, support = 1) to remove noise, and a fixed threshold of 220 was used to extract the mineralized bone from soft tissue and marrow phase. The reconstruction and 3D quantitative analyses were performed by using software provided by Scanco. The same settings for scan and analysis were used for all samples.

For distal femoral trabecular bone analyses, the scans were initiated from the growth plate cross-sectionally and moved proximally for a total of 300 slices. From this region, a region of interest (ROI) that consisted of 100 slices starting from about 0.5 mm proximal to growth plate, constituting 1.6 mm in length, was chosen for analyses. Cancellous bone was separated from the cortical regions by semi-automatically drawn contours. The following 3D indices in the defined ROI were analyzed: bone volume (BV, mm^3^), tissue (cortical and marrow) volume (TV, mm^3^), relative bone volume over total volume (BV/TV, %), trabecular number (Tb.N, mm^-1^), trabecular thickness (Tb.Th, µm), trabecular separation (Tb.Sp, µm), connectivity density (Conn.Dn, mm^-3^), structure model index (SMI, ranges from 0 to 3 with 0 = platelike and 3 = rodlike), and bone mineral density (BMD, g hydroxyapatite/cm^3^). BMD is the average density of the segmented fraction of the ROI (bone) not including the marrow cavity.

*5. Dynamic histomorphometry*

The distal femurs were cut in 6 μm thickness of sections with longitudinal 4-10 μm by microtome equipped with a tungsten-carbide knife. Both segments of femur mid-shaft and distal femur parts were dehydrated in graded alcohols, cleared in xylene, and embedded in Methyl Methacrylate (MMA) plastic. Using the diamond-embedded wire saw at femur mid-shaft, three consecutive transverse sections were cut approximately at a 120 μm thickness. Sections were ground to the desired thickness of 20-30 μm using 600 grit sand paper. Sections were mounted on microscope slides, cleared in xylenes, and cover-slipped with Eukitt. By using the unstained sections which maintains flurochrome in newly mineralized bone, the primary data were measured from the distal femur trabecular bone region 0.4 mm distal to growth plate and 0.5 mm away from intracortical surface. Under OLYMPUS fluorescence microscope, using OsteoMeasure™ system, at 200 X magnification: Tissue Area (T.Ar), Bone Area (B.Ar), total perimeter (B.Pm), single labeled perimeter (sL.Pm), double labeled perimeter (dL.Pm), and inter-labeled width (Ir.L.Th) of trabecular bone surface were generated as primary data. These primary data were produced by tracing and measuring the area of trabecular bone region of interest (Tissue Area), individual trabecular bone perimeter (B.Pm) and area (Bone Area), single fluorescent dye labeled perimeter (sL.Pm), double fluorescent dyes labeled overlaying perimeters (dL.Pm), and the distance between two flurochrome labels or also called inter-labeled width (Ir.L.Th). There were 4 days apart between two fluorescent labels. From these primary data the quantified data were derived: Bone area/tissue area (BV/TV) = B.Ar/T.Ar (%), MS/BS = (dL.Pm+1/2sL.Pm)/B.Pm×100 (%), MAR = Ir.L.Th / 4 days (μm/day), and BFR/BS = MAR × MS/BS × 365 (μm^3^/μm^2^/year) [12, 13].

*6. Bone TRAP staining*

In order to quantify the osteoclast number and activity, Tartrate-acid Resistant Acid Phosphatase (TRAP) stain was used to specifically label osteoclasts. Distal femur sections were deplastified in acetone and then rehydrated in graded alcohols and distilled water (ddH_2_O). Slides were then pre-incubated in 0.2 M acetate buffer (pH 5.0) (CAS 127-09-3; FW 82.03; Sigma-Aldrich 241245) with 50 mM sodium tartrate dibasic dehydrate(CAS 6106-24-7; FW 230.08; Sigma-Aldrich T6251) for 20 minutes at RT. Slides were transferred (drained briefly) from pre-incubation to incubation media with 0.2M acetate buffer (pH 5.0), napthol AS-MX phosphate (0.5 mg/ml) (CAS 1596-56-1; FW 371.3; Sigma-Aldrich N4875), and fast red TR salt (1.1 mg/ml) (CAS 51503-28-7; FW 440.90; Sigma-Aldrich F6760) for 2 hours in 37 °C water bath. Then incubation media was discarded and slides were covered with ddH_2_O for 5 minutes at RT. Slides were counterstained by Toluidine Blue (Sörensen’s Buffer). Finally, all slides were coverslipped with aqueous-based mounting media.

To quantify osteoclasts activity, under bright field of OLYMPUS fluorescence microscope, using OsteoMeasure System™ (Osteometrics, Atlanta, GA, USA), at 400 X magnification, the primary data were measured from the distal femur trabecular bone region 0.4 mm distal to growth plate and 0.5 mm away from intracortical surface (region of interest). By tracing the total area of region of interest, trabecular bone area and perimeter, and osteoclast covered surface, counting osteoclast numbers in the region of interest trabecular bone surface, Tissue Area (T.Ar), Bone Area (B.Ar), total perimeter (B.Pm), Osteoclast number (N.Oc), and Osteoclast surface (Oc.S) were measured as primary data. From those primary data the quantified data were derived: Bone Volume/Tissue Volume (BV/TV) = B.Ar/T.Ar (%), Osteoclast surface/Trabecular Bone surface (Oc.S.BS) = Oc.S/B.Pm (%), and Osteoclast number/Trabecular Bone surface (N.Oc/BS) = N.Oc/B.Pm (#/mm) [12, 14].

*7. Ex vivo Osteoclastogenesis and Osteoblastogenesis Assays*

Procedures for study of osteoblast and osteoclast formation from bone marrow stem cells were followed the methods previously described [1, 2, 10]. Briefly, to study osteoclast formation, bone marrow cells were isolated from the femora and tibiae in α-MEM. Marrow cells were rinsed and resuspended in α-MEM then seeded into wells using an equal number of cells in each well and were cultured for 2 days in α-MEM supplemented with human macrophage colony-stimulating factor (M-CSF; 5 ng/ml). The nonadherent cells were collected and purified by Ficoll-Plus (Amersham Pharmacia Biotech Inc., Arlington Height, IL, USA) then seeded into wells, again with an equal number of cells per plate, and incubated in α-MEM containing M-CSF (30 ng/ml) and RANKL (60 ng/ml) for 5 days. Osteoclasts were identified by staining for TRAP using a kit (Sigma-Aldrich, St. Louis, MO). The number of TRAP-positive multinucleated cells was then counted; a parallel set of osteoclast cultures at day 5 was used to extract total RNA for microarray analysis as described below.

To study osteoblast formation, bone marrow cells were cultured in α-MEM supplemented with 15% preselected FCS (Hyclone, Logan, UT, USA) and ascorbic acid-2-phosphate (1 mM). At 10 days, the recruitment of mesenchymal cell progenitors into the osteoblastic lineage was assessed by counting the number of alkaline phosphatase-positive colonies (CFU-F) (Sigma); a parallel set of osteoblast cultures at day 10 was used to extract total RNA for microarray analysis as described below. In separate plates, culture was continued until day 28, when the number of colonies producing mineralized bone matrix (CFU-OB) was determined after von Kossa staining.

*8. Quantitative PCR*

Total RNA from ex vivo cultured osteoblasts and osteoclasts was used for real time PCR determination of mRNA levels as described previously. Briefly, total RNA was first enriched on RNAeasy columns followed by digestion of any carried over genomic DNA using DNAse using the RNeasy Plus mini kit (Qiagen,Valencia, CA, USA).Total RNA then was quantified and assessed for RNA integrity using an Agilent 2100 Bioanalyzer. 1 µg of total RNA was used for reverse transcription to generate a cDNA library with a High-Capacity cDNA Reverse Transcription Kit from Applied Biosystems (Forster City, CA). After 1:25 dilution of libraries into water, mRNA levels for specific mRNAs were determined by real time PCR using an ABI 7500 real time PCR machine and Taqman Assay On Demand probesets obtained from Applied Biosystems. Each sequence of the probesets is available on the Applied Biosystems’s website. Changes in gene expression were calculated using the 2^-∆∆Ct^ method using 18S RNA as the internal control, and the Sham group as the control.

**Supplementary Figure Legends**

**Fig. 1. Effect of NP159 on cortical architecture of the femur midshaft as assessed by micro-CT. (A)** Representative 3D images of cortical microarchitecture. Measurement are shown for: **(B)** Cortical thickness (Ct.Th, µm), and **(C)** Bone stiffness (N/mm) by finite element analysis, respectively. Data are expressed as mean ± SEM. N=10 per group. Significance of differences was determined using one-way ANOVA with a Newman–Keuls test post hoc. *P < 0.05 and **P < 0.01 versus the indicated group. NS, no significant difference.

**Fig. 2. Effects of NP159 on levels of serum biomarkers of bone.** Serum concentrations of osteocalcin were measured using a rat osteocalcin immunoassay kit (Alfa Aesar). Data are expressed as mean ± SEM, N = 10 to 13 per group. Significance of differences was determined using one-way ANOVA with a Newman–Keuls test post hoc. NS, no significant difference.

Supplemental Figure 1

**B**

**C**


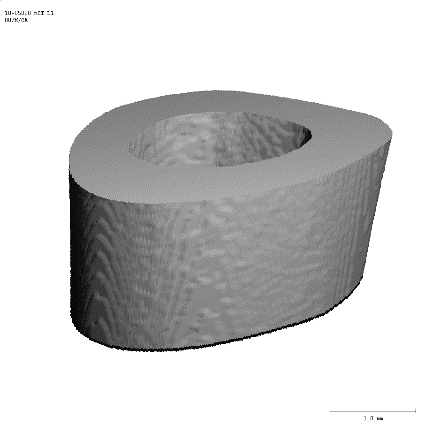

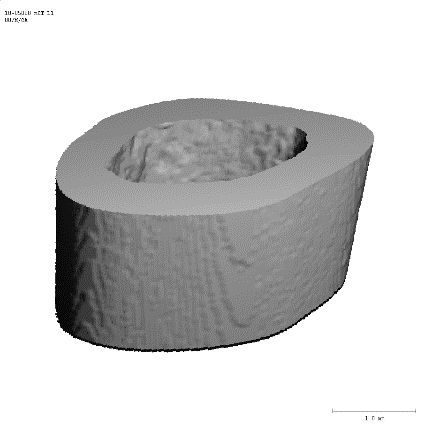

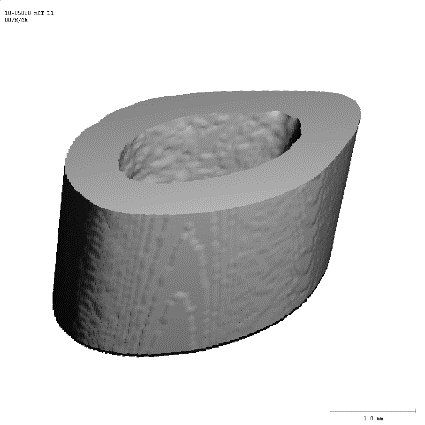


**A**

**Sham**

**SCI**

**SCI+NP159**

Supplemental Figure 2

**Reference:**

1. Sun, L., et al., *Anabolic steroids reduce spinal cord injury-related bone loss in rats associated with increased Wnt signaling.* J Spinal Cord Med, 2013. **36**(6): p. 616-22.

2. Qin, W., et al., *The Central Nervous System (CNS)-independent Anti-bone-resorptive Activity of Muscle Contraction and the Underlying Molecular and Cellular Signatures.* J Biol Chem, 2013. **288**(19): p. 13511-21.

3. Zhao, W., et al., *Electrical stimulation of hindlimb skeletal muscle has beneficial effects on sublesional bone in a rat model of spinal cord injury.* Bone, 2021. **144**: p. 115825.

4. Zhao, W., et al., *Sclerostin Antibody Reverses the Severe Sublesional Bone Loss in Rats After Chronic Spinal Cord Injury.* Calcif Tissue Int, 2018. **103**(4): p. 443-454.

5. Peng, W., et al., *Systemic administration of an antagonist of the ATP-sensitive receptor P2X7 improves recovery after spinal cord injury.* Proc Natl Acad Sci U S A, 2009. **106**(30): p. 12489-93.

6. Peng, Y., et al., *Administration of High-Dose Methylprednisolone Worsens Bone Loss after Acute Spinal Cord Injury in Rats.* Neurotrauma Rep, 2021. **2**(1): p. 592-602.

7. Qin, W., et al., *Sclerostin antibody preserves the morphology and structure of osteocytes and blocks the severe skeletal deterioration after motor-complete spinal cord injury in rats.* J Bone Miner Res, 2015. **30**(11): p. 1994-2004.

8. Qin, W., et al., *Mice with sclerostin gene deletion are resistant to the severe sublesional bone loss induced by spinal cord injury.* Osteoporos Int, 2016. **27**(12): p. 3627-3636.

9. Cardozo, C.P., et al., *Nandrolone slows hindlimb bone loss in a rat model of bone loss due to denervation.* Ann N Y Acad Sci, 2010. **1192**: p. 303-6.

10. Bramlett, H.M., et al., *Effects of low intensity vibration on bone and muscle in rats with spinal cord injury.* Osteoporos Int, 2014. **25**(9): p. 2209-19.

11. Cao, J.J., B.R. Gregoire, and H. Gao, *High-fat diet decreases cancellous bone mass but has no effect on cortical bone mass in the tibia in mice.* Bone, 2009. **44**(6): p. 1097-104.

12. Zhou, H., et al., *Osteoblast/osteocyte-specific inactivation of Stat3 decreases load-driven bone formation and accumulates reactive oxygen species.* Bone, 2011. **49**(3): p. 404-11.

13. Chandrasekhar, K.S., et al., *Blood vessel wall-derived endothelial colony-forming cells enhance fracture repair and bone regeneration.* Calcif Tissue Int, 2011. **89**(5): p. 347-57.

14. Erben, R.G., *Embedding of bone samples in methylmethacrylate: an improved method suitable for bone histomorphometry, histochemistry, and immunohistochemistry.* J Histochem Cytochem, 1997. **45**(2): p. 307-13.
